# Simulation of AMPA and NMDA contribution to postsynaptic response

**DOI:** 10.1186/1471-2202-16-S1-P91

**Published:** 2015-12-18

**Authors:** Crhistian Miguel Gutiérrez Galindo, Virginia González Vélez, Amparo Gil

**Affiliations:** 1 Dept. Ciencias Básicas, Universidad Autónoma Metropolitana Azcapotzalco, 02200, México DF, México; 2 Dept. MACC, Universidad de Cantabria, 39005, Santander, Spain

## 

**Figure 1:**
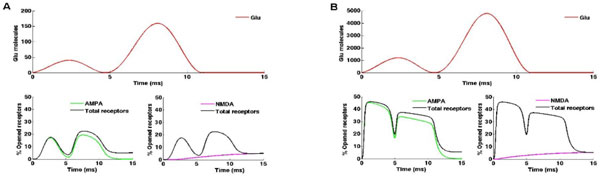
**Simulations **A **Low-concentration pulses**. **B **High-concentration pulses. Above input trains (red) and below AMPA (green), NMDA (magenta) and Total receptors (black) responses.

The synaptic response depends on the dynamics of the released neurotransmitter, as well as on the ability of ionic receptors (AMPA and NMDA) to activate and deactivate in time. Simulation of these receptors allow to study interesting patterns as neuronal plasticity, which modulates the efficiency of transmission in two different manners: facilitation (incremented response to weak stimuli), and depression (decreased response to strong stimuli). In this work, we have simulated the postsynaptic response due to AMPA and NMDA activation. Our model considers the postsynaptic membrane as a disk of 0.1 microns of radius where receptors lie with a ratio of 4:1 (80 AMPA and 20 NMDA) [1]. They are uniformly distributed in the whole area of the disk, assuming they have the same probability of being activated as they come in contact with neurotransmitter molecules which are dispersed homogeneously over the postsynaptic membrane. We use the kinetic models proposed by [2] and [3] for AMPA and NMDA receptors, respectively. The model was solved with a stochastic simulation algorithm, and plots show the mean values calculated from 500 runs. Figures 1A and 1B show the results obtained when the neurotransmitter comes in the form of two pulses of different concentrations in a short period of time. Figure 1A corresponds to the case of low concentration pulses (40 and 160 molecules for the first and second pulses, respectively). In this simulation, it can be seen how AMPA receptors increase their response to the second stimulus, exhibiting facilitation. Figure 1B shows the case of high concentration pulses (1207 and 4787 molecules, respectively). In this simulation, a desensitized response is observed, that is, a depression effect which reduces the number of open receptors for the second stimulus. In both cases it is observed that the contribution made by the NMDA receptors is identical, whereas AMPA receptors have a contribution that follows the rapid dynamics of the neurotransmitter released by the presynaptic neuron.

Other simulations for different trains of neurotransmitter were performed, and similar results were obtained. We also performed simulations for different stimulation frequencies in larger timescales and again, AMPA receptors followed rapid neurotransmitter dynamics while NMDA receptors exhibited changes only in time activation. Then, we conclude that the contribution of AMPA receptors is the most important one in short timescales, whereas NMDA receptors participate mainly in larger timescale responses. These results are the basis of our current work on proposing a postsynaptic model intended to work with our presynaptic model [4].
